# The Incidence of Spontaneous Bacterial Peritonitis in Patients With Cirrhosis-Related Ascites Undergoing Elective Outpatient Large-Volume Paracentesis

**DOI:** 10.7759/cureus.50191

**Published:** 2023-12-08

**Authors:** Arteen Arzivian, Tuan Duong

**Affiliations:** 1 Gastroenterology and Hepatology, Wollongong Hospital, Wollongong, AUS

**Keywords:** chronic liver disease, decompensated cirrhosis, massive ascites, large volume paracentesis, spontaneous bacterial peritonitis (sbp)

## Abstract

Background and aims

Large-volume paracentesis (LVP) is a common practice for diuretic-refractory ascites in patients with cirrhosis. Spontaneous bacterial peritonitis (SBP) is reportedly low in asymptomatic patients presenting for elective outpatient LVP. The benefits and cost-effectiveness of routine testing for SBP in these patients are yet to be established. We aimed to investigate the incidence of SBP in outpatients who underwent elective LVP and the diagnostic yield of routine ascitic fluid testing, specifically fluid culture, and to assess the cost-effectiveness of these tests.

Methods

This is a retrospective study of adult patients undergoing outpatient LVP at Wollongong Hospital over three years. Symptomatic patients and patients with non-cirrhosis-related ascites were excluded. The ascitic fluid results were reviewed to determine if the polymorphonuclear (PMN) cell count was above 250 x 10^6^/L and whether this was associated with positive fluid culture and clinical outcomes. The primary outcome was the incidence of SBP. The secondary outcome was the incidence of bacterascites.

Results

There were 26 patients with 122 elective ascitic taps. Two of 122 taps (1.6%) had ascitic PMN count above 250 x 10^6^/L, indicating SBP. Four out of 122 taps (3.2%) had positive ascitic fluid cultures with a normal PMN count. All patients did not have significant clinical outcomes, did not receive antibiotic treatment, and remained asymptomatic over three years of follow-up.

Conclusions

Routine testing of ascitic fluid cell count and culture in asymptomatic patients with cirrhosis presenting for outpatient LVP yielded a low incidence of SBP and bacterascites, which were not clinically significant. Routine testing is costly and potentially exposes patients to antibiotics unnecessarily.

## Introduction

Chronic liver disease (CLD) affects more than 300 million people worldwide; it is mainly caused by metabolic-associated steatotic liver disease (MASLD) secondary to obesity and diabetes mellitus, excessive alcohol consumption, and viral hepatitis; this number is expected to continue rising with the increasing prevalence of obesity and related metabolic risk factors. Patients with advanced chronic liver disease (ACLD) or cirrhosis are characterized by a more severe degree of fibrosis, liver architectural distortion, and synthetic dysfunction; these patients usually progress from a “compensated” phase to a “decompensated” phase over a long period. During the “compensated” phase, patients are generally asymptomatic and do not have specific clinical signs indicative of cirrhosis; they might have non-specific symptoms (e.g., lethargy) or biochemical and radiological features suggesting the diagnosis. On the other hand, patients with “decompensated” cirrhosis present with multiple complications related to liver dysfunction, including ascites, bleeding from gastroesophageal varices, spontaneous bacterial peritonitis (SBP), hepatorenal syndrome, and hepatic encephalopathy [1]. SBP is associated with high mortality and leads to cirrhosis being the fourth cause of death in adults in Western countries [2].

SBP is the most common bacterial infection in patients with cirrhosis and ascites; it is suspected when patients develop abdominal pain, fever, and sepsis. It can also have non-specific symptoms such as lethargy and confusion as well as precipitate other cirrhosis complications; the diagnosis is confirmed when the ascitic fluid polymorphonuclear count is above 250 x 10^6^/L. Gram-negative enteric organisms (e.g., *Escherichia coli* or *Klebsiella pneumoniae*) comprise the majority of the causative microorganisms, suggesting that the gastrointestinal tract is the primary source of contamination and infection [3,4]. SBP has a prevalence rate of 10%-30% in patients with cirrhosis, and a recent epidemiological meta-analysis showed a pooled prevalence of 17%. SBP is associated with multiple complications, including acute kidney injury, hepatic encephalopathy, and a doubling of the mortality rate compared to patients with cirrhosis who do not have this complication [5].

Due to this high prevalence and associated mortality, performing a diagnostic paracentesis for all hospitalized patients or patients presenting to the emergency department with symptoms and findings suggestive of SBP is recommended [6,7]. The ascitic fluid should be sent for a diagnostic work-up, including a differential cell count, serum-ascites albumin gradient (SAAG), and ascitic fluid culture. Other tests, including ascitic fluid amylase level, fluid cytology, flow cytometry, and culture for mycobacterium, may be needed depending on the clinical scenario [8].

“Refractory ascites” describes ascites that cannot be controlled and managed by diet and the use of diuretics; it requires regular large-volume paracentesis (LVP) or the creation of trans-jugular intrahepatic portosystemic shunts (TIPSS) [9]. In asymptomatic outpatients presenting for elective LVP, the incidence of SBP is reported to be less than that in symptomatic patients; this lower incidence is also associated with lower morbidity and mortality [5]. Despite this, routine testing of ascitic fluid in this subset of patients continues to be a routine practice by most practitioners. There is a paucity of evidence and a lack of guidance regarding this topic, with both the American Society of the Study of Liver Disease (AASLD) guideline in 2021 [6] and the European Association of the Study of the Liver (EASL) guideline in 2018 [7] not addressing this issue. A meta-analysis studied this specific clinical question, showed SBP to have a very low incidence and prevalence in this setting, and raised questions regarding the benefit of routine testing of ascitic fluid for these patients [10].

Furthermore, routine ascitic fluid testing in the outpatient setting leads to complex decision-making with asymptomatic patients having positive results; it also leads to unnecessary antibiotic exposure, consequently leading to multiresistant organisms. It is unclear if this practice is cost-effective and likely to cause avoidable waste of resources and increase total cirrhosis-related health expenditure.

We face this clinical scenario daily with the associated uncertainty regarding further management; we conducted this retrospective study to confirm that SBP in this setting is of low incidence in our facility and routine analysis of ascitic fluid is associated with unnecessary costs without associated benefits for patients.

## Materials and methods

This was a retrospective study conducted by the Gastroenterology Department at Wollongong Hospital for patients presenting to the ambulatory care unit to undergo an elective outpatient LVP. Ethics approval was gained, and the Illawarra Health and Medical Research Institute (IHMRI) ethics committee approved the study. It was performed by the ethical standards laid down in the Declaration of Helsinki. Considering the retrospective nature of the study, consent from patients was deemed unnecessary. All patients’ identity-related data were de-identified during the period of the study and writing of the manuscript.

Data were collected from the ambulatory care unit records; it covered the period between January 2019 and January 2022. During this time, the elective outpatient ascitic tap rate was affected by COVID-19-related restrictions. The Wollongong Hospital and Gastroenterology Department’s electronic medical records were reviewed for patients’ clinical details and ascitic fluid investigation results. We included adult patients (above the age of 18 years) with cirrhosis secondary to any etiology complicated by refractory ascites requiring regular LVP. Data was meticulously observed to confirm that patients in the study were asymptomatic when the ascitic fluid analysis was performed. Patients with symptoms attributed to SBP and those with ascites secondary to other causes (e.g., malignancy) were excluded.

We extracted and documented different variables for each patient at the time of the ascitic fluid analysis; these variables included age, sex, etiology of cirrhosis, Child-Pugh score, and model for end-stage liver disease (MELD)-Na score (around the time of the tap), history of previous SBP, use of prophylactic antibiotics, ascitic fluid polymorphonuclear (PMN) count, RBC count (only if PMN count above 250 x 10^6^/L), and fluid culture results. Other variables extracted and gathered but deemed non-essential to be analyzed included the ongoing use of diuretics, beta-blockers, proton pump inhibitors, renal function, and ascitic fluid color (not used due to subjectiveness).

The primary outcome of the study was the incidence of SBP, defined as a PMN count above 250 x 10^6^/L. The secondary outcome was “bacterascites,” a positive ascitic fluid culture with a PMN count of less than 250 x 10^6^/L. These outcomes were chosen due to their direct association with poor prognosis and high mortality [7,11].

Pearson’s correlation coefficient (Pearson’s r) and independent samples t-test were used to correlate between variables. A p-value of <0.05 was considered statistically significant. Statistical Package for the Social Sciences (SPSS) v28 (IBM Corp., Armonk, NY) was used for statistical analysis.

## Results

**Figure 1:**
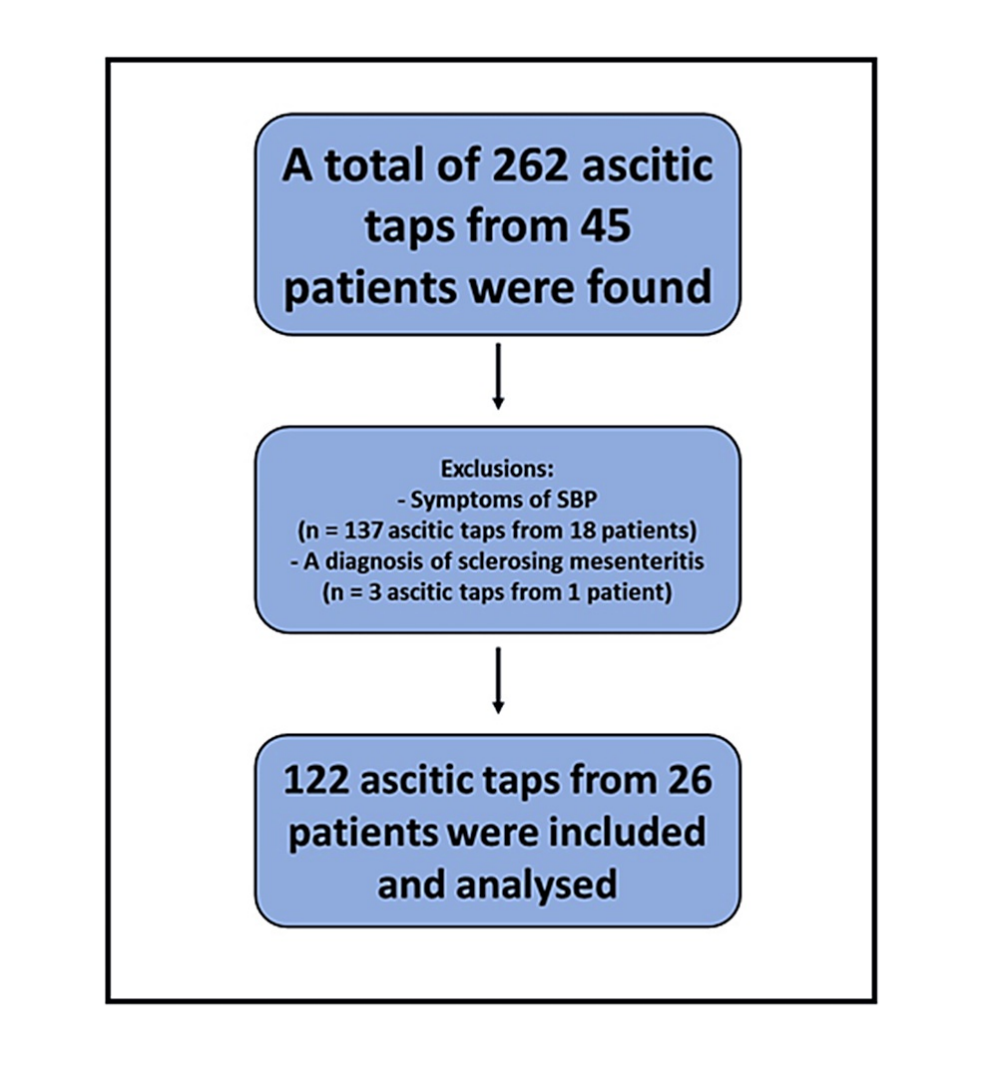
The selection process of eligible paracentesis episodes included in the study Most exclusions were due to symptoms that could be associated with SBP at the time of the paracentesis; the most common symptoms were abdominal pain, followed by fever and then lethargy. One patient with three paracentesis episodes was excluded due to non-cirrhosis-related ascites (sclerosing mesenteritis). SBP: Spontaneous bacterial peritonitis.

The total number of paracentesis episodes found in the records was 262 from 45 patients. After applying the inclusion and exclusion criteria, the total number of paracentesis episodes eligible to be analyzed was 122 from 26 patients (mean of 4.7 paracentesis episodes per patient); most episodes excluded were due to symptoms that could be associated with SBP at the time of the paracentesis; most common symptoms were abdominal pain, followed by fever then lethargy. One patient with three paracentesis episodes was excluded due to non-cirrhosis-related ascites (sclerosing mesenteritis). Figure 1 illustrates the process of choosing the number of ascitic taps eligible for the study.

The median age of patients was 65 years (minimum age of 47 and maximum age of 90) with a male:female ratio of 22:4. Alcoholic liver disease was the most common etiology (42%), followed by chronic hepatitis C (12%), MASLD (8%), and chronic hepatitis B, and multifactorial etiology was found in 10 of the 26 patients (38%). Most patients had a Child-Pugh B score and a MELD-Na score between 10 and 19 at their paracentesis. Patients with a history of SBP were four out of 26 (15%), and only two out of those four patients were on prophylactic antibiotics (norfloxacin and trimethoprim/sulfamethoxazole). Both patients had no recurrent SBP or growth of ascitic fluid during the study period. Most of the analyzed ascitic fluids did not report albumin levels; hence, they were not included in the extracted variables.

**Figure 2:**
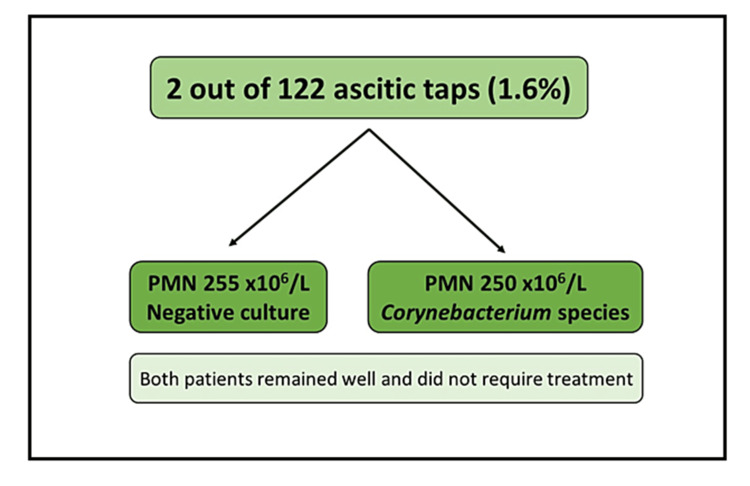
The primary outcome (spontaneous bacterial peritonitis) defined as a PMN count of 250 x 10^6/L or above PMN: Polymorphonuclear count.

The primary outcome, SBP, was found in two paracentesis episodes from two patients (1.6% of the total samples analyzed). The first patient had a polymorph count of 255 x 10^6^/L with an RBC count of 245 x 10^6^/L, and the ascitic fluid culture was negative. The second patient had a polymorph count of 250 x 10^6^/L with an RBC count of 5150 x 10^6^/L, and the culture grew *Corynebacterium* species. Both patients had no history of SBP at the time of the elective paracentesis; both remained asymptomatic over three years of follow-up and required no antibiotics or further management. Of note, seven patients had no polymorphonuclear cell count reported, and none of these seven patients had positive ascitic fluid cultures. Figure 2 illustrates the primary outcome results.

**Figure 3:**
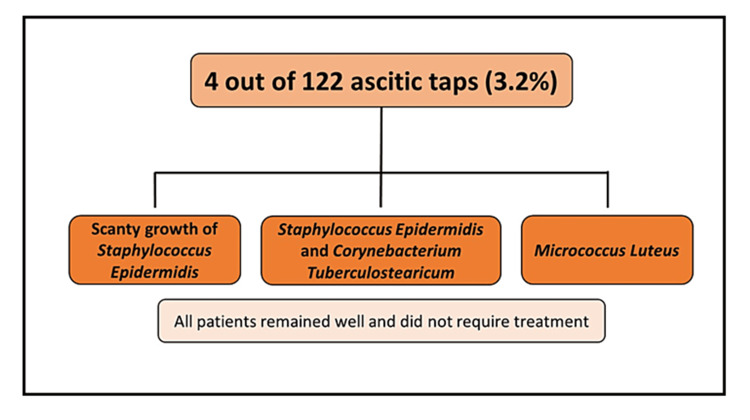
The secondary outcome (bacterascites) defined as a positive ascitic fluid culture with a PMN count of less than 250 x 10^6/L PMN: Polymorphonuclear count.

The secondary outcome, bacterascites, was found in four paracentesis episodes from three patients (3.2% of the total samples analyzed). One patient had a scanty growth of *Staphylococcus epidermidis*, the second had *Micrococcus luteus* growth, and the third had *Staphylococcus epidermidis* on one sample and *Corynebacterium tuberculostearicum* on a different sample. All patients were asymptomatic, remained well, and did not require treatment for these episodes. Figure 3 illustrates the secondary outcome results.

There was no statistically significant correlation between the Child-Pugh score, MELD-Na score, etiology of cirrhosis, previous SBP, medications used, and the primary or secondary outcomes (p-value > 0.05). Table 1 summarizes the characteristics and outcomes of the study.

**Table 1:** Results and characteristics of patients (total number = 26 patients) ^† ^This is the average age, Child-Pugh and MELD-Na scores over the three years of the study. ^‡^ Both patients were on norfloxacin. ^§^ Methotrexate (MTX) was used for psoriasis. ^¶^ MELD-Na could not be calculated due to the unavailability of sodium level. * The elevated polymorph/RBC count and the growth of *Staphylococcus epidermidis* are on two different samples. Note: All cell counts are in x10^6^/L units. SBP: Spontaneous bacterial peritonitis; PMN: Polymorphonuclear count; MASLD: Metabolic-associated steatotic liver disease; EtOH: Ethanol; Hep: Hepatitis; MTX: Methotrexate; N/A: Not applicable.

Patient	No. of taps	Age (years)*	Sex	Cause of cirrhosis	Child-Pugh^†^	MELD-Na*	Previous SBP	Antibiotics^‡^	Polys > 250	RBCs	Positive culture
1	7	69	F	MASLD	B	10-19	No	No	250	5150	*Corynebacterium *species
2	4	59	M	EtOH	B	10-19	No	No	No	N/A	No
3	1	71	M	EtOH	B	10-19	No	No	No	N/A	No
4	1	56	M	EtOH + HepC	C	20-29	No	No	No	N/A	No
5	2	54	M	EtOH + HepC	B	10-19	No	No	No	N/A	No
6	15	57	M	EtOH + HepC	B	10-19	Yes	No	255*	245*	Scanty growth of Staphylococcus epidermidis*
7	1	52	M	EtOH + HepC + HepB	B	10-19	No	No	No	N/A	No
8	5	66	F	EtOH	C	20-29	No	No	No	N/A	Twice (*Staphylococcus epidermidis *and *Corynebacterium tuberculostearicum*)
9	14	77	M	EtOH	B	10-19	Yes	Yes	No	N/A	No
10	3	61	M	EtOH + HepC	B	10-19	No	No	No	N/A	No
11	3	86	M	EtOH + MTX^§^	B	10-19	No	No	No	N/A	No
12	2	63	M	HepC	C	20-29	No	No	No	N/A	No
13	1	61	M	EtOH	C	N/A^¶^	No	No	No	N/A	No
14	16	74	M	EtOH	B	10-19	Yes	No	No	N/A	Micrococcus luteus
15	10	68	M	EtOH	B	10-19	No	No	No	N/A	No
16	18	68	M	MASLD	B	10-19	Yes	Yes	No	N/A	No
17	1	90	M	EtOH	B	10-19	No	No	No	N/A	No
18	1	51	M	EtOH	C	20-29	No	No	No	N/A	No
19	4	62	M	EtOH + HepC	C	10-19	No	No	No	N/A	No
20	2	47	F	EtOH	C	20-29	No	No	No	N/A	No
21	1	65	M	HepC	B	20-29	No	No	No	N/A	No
22	4	64	M	EtOH + HepC	B	10-19	No	No	No	N/A	No
23	2	75	M	EtOH	C	10-19	No	No	No	N/A	No
24	1	56	M	EtOH + HepC	C	20-29	No	No	No	N/A	No
25	1	61	M	HepC	C	N/A^£^	No	No	No	N/A	No
26	2	83	F	HepB + MASLD	C	20-29	No	No	No	N/A	No

## Discussion

Ascites is the most common symptomatic manifestation of decompensated chronic liver disease [12], associated with a higher incidence of SBP and increased morbidity and mortality. These higher adverse outcomes seem to be mainly related to hospitalized patients presenting with acute complications of decompensation, including gastroesophageal varices bleeding, hepatorenal syndrome, and hepatic encephalopathy [13,14]. In asymptomatic outpatients who require regular LVPs to manage refractory ascites, the incidence of SBP is reported to be low, and the clinical benefit and cost-effectiveness of routine ascitic fluid testing in this setting are questionable [15,16].

Our study showed a low incidence of SBP and bacterascites in asymptomatic patients presenting for outpatient LVP. SBP was found in 1.6% of the LVPs analyzed; this low rate is consistent with other studies reported in the literature [17]. Bacterascites were found in 3.2% of the cohort; there was no growth of typical gram-negative microorganisms associated with clinically significant SBP [18]; the organisms grown in our cohort were more consistent with sample contamination, which suggests that the actual rate of bacterascites is even lower. Despite no specific treatment, there were no clinically significant outcomes in this small percentage of patients who met the definition of SBP or bacterascites. Our study results are consistent with previous individual studies and a meta-analysis by Alotaibi et al., which showed that the incidence of SBP in asymptomatic outpatients with decompensated cirrhosis requiring LVP is low and raised a question regarding the benefit of routine analysis of all paracentesis samples in this population [10].

We also demonstrated that regular and routine investigations of ascitic fluid in this setting are associated with costs. The costs of investigations were requested from the pathology department at our hospital as per the Australian Medicare Benefits Scheme. Measuring ascitic fluid albumin level costs 9.7 Australian Dollars (AUDs), cell count costs 12.5 AUDs, culture costs 48.15 AUDs, and an administration fee of approximately 17 AUDs was collected for each sample received; this collectively costs 87 AUDs for each sample processed, which corresponds to a cost of more than 6,000 AUDs throughout the study. These costs do not include the labor to handle the samples, the time to process them, and other related expenses, which we believe cost more than the crude cost of the investigations, especially in a resource-limited healthcare system in the post-COVID-19 era. This cost of routine investigations is compared to the cost of SBP treatment and prophylaxis. According to the Australian Pharmaceutical Benefits Scheme (PBS), the dispensed price for a maximum quantity (DPMQ) of intravenous ceftriaxone 2 g for five days for SBP treatment is 26.81 AUDs, and DPMQ for ongoing SBP prophylaxis with once daily trimethoprim 160 mg + sulfamethoxazole 800 mg is approximately 47 AUDs monthly.

The strengths of our study include excluding patients with any symptoms that could be related to SBP; this ensures only asymptomatic patients were studied. We demonstrated that the outcomes are regardless of age, sex, and cause of liver disease. We also calculated Child-Pugh and MELD-Na scores for the patients in the study. We confirmed that it does not affect the low incidence of SBP in this group of patients.

Limitations in our study include the relatively low number of patients and seven ascitic fluid samples that did not have a polymorphonuclear cell count reported, and most ascitic fluid albumin levels were unavailable. The ascitic fluid albumin level would have been necessary to demonstrate if a low albumin level is associated with an increased risk of SBP in future paracentesis, which has been reported and recommended as an indication for primary antibiotic prophylaxis [19].

## Conclusions

In asymptomatic patients with cirrhosis presenting for outpatient LVP, routine testing of ascitic fluid cell count and culture yields a low incidence of SBP and bacterascites, which were not clinically significant. Routine testing in this setting is associated with costs and potentially exposes patients to antibiotics unnecessarily. Hence, routine testing is not recommended.
